# Testing the role of intraflagellar transport in flagellar length control using length-altering mutants of *Chlamydomonas*

**DOI:** 10.1098/rstb.2019.0159

**Published:** 2019-12-30

**Authors:** Kimberly Wemmer, William Ludington, Wallace F. Marshall

**Affiliations:** Department of Biochemistry and Biophysics, University of California San Francisco, San Francisco, CA 94158, USA

**Keywords:** *Chlamydomonas*, intraflagellar transport, organelle size control

## Abstract

Cilia and flagella are ideal model organelles in which to study the general question of organelle size control. Flagellar microtubules are steady-state structures whose size is set by the balance of assembly and disassembly. Assembly requires intraflagellar transport (IFT), and measurements of IFT have shown that the rate of entry of IFT particles into the flagellum is a decreasing function of length. It has been proposed that this length dependence of IFT may be the basis for flagellar length control. Here, we test this idea by showing that three different long-flagella mutations in *Chlamydomonas* all cause increased IFT injection, thus confirming that IFT can influence length control. However, quantitative comparisons with mathematical models suggest that the increase in injection is not sufficient to explain the full increase in length seen in these mutants; hence, some other mechanism may be at work. One alternative mechanism that has been proposed is length-regulated binding of tubulin to the IFT particles. However, we find that the apparent length dependence of tubulin loading that has previously been reported may actually reflect length-dependent organization of IFT trains.

This article is part of the Theo Murphy meeting issue ‘Unity and diversity of cilia in locomotion and transport’.

## 1. Introduction

A long-standing but unanswered question in cell biology is what mechanisms determine the size of organelles [1]. For most organelles, size is inherently difficult to measure because the organelles have complicated three-dimensional structures and are located deep within the cell where imaging can be difficult. Flagella, by contrast, are simple to visualize and, because the only size parameter of a flagellum that can vary is their length, they represent a one-dimensional instance of the more general organelle size control problem [2].

**Figure 1.**
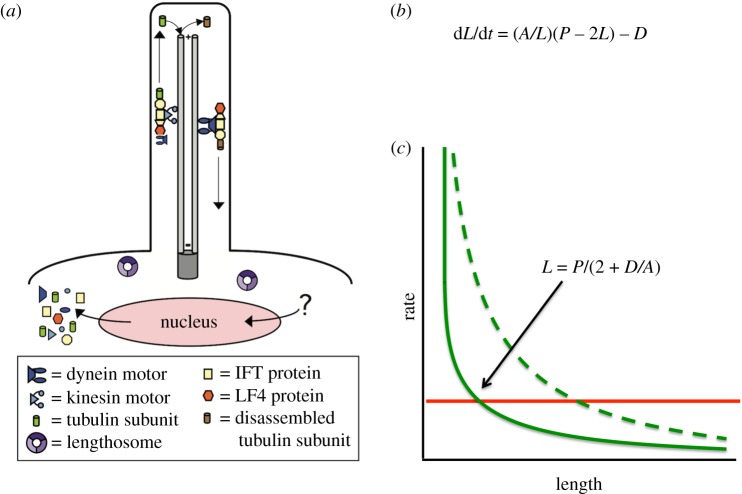
Flagellar length control and IFT. (*a*) Diagram of flagellar dynamics. IFT particles consisting of IFT proteins plus motors assemble at the base of the flagellum, recruit cargo such as tubulin subunits and move to the flagellar tip. At the tip, cargo is delivered, and products of flagellar disassembly are removed. Anterograde motion to the tip is powered by kinesin-2, while retrograde motion back to the cell body is powered by dynein. Also shown in this diagram are two key regulators of flagellar length—the cytoplasmic ‘lengthosome’ complex and the flagellar LF4 kinase. Both the entry of IFT into flagella and the loading of tubulin and other cargo onto the IFT trains have been proposed to be length-dependent. As indicated in the diagram, unknown factors regulate transcription of cargo proteins during flagellar assembly. (*b*) Flagellar length changes at a rate that depends on the difference of a growth term and a disassembly term. The growth rate is proportional to the rate of IFT injection, previously shown to scale as 1/*L*. Growth rate is also proportional to the available free concentration of flagellar precursor proteins in the cytoplasm, which is taken as a total pool *P* minus the quantity of protein incorporated into the two flagella of a biflagellate cell. (*c*) Flagellar length as the steady-state balance between length-dependent assembly (green) and length-independent disassembly (red). The steady-state solution is unique and given by the expression shown. Mutations that increase IFT by increasing the proportionality constant *A* in the equation result in a new assembly curve (dashed line) that intersects the disassembly at a longer steady-state length.

Flagella (also known as cilia) are protrusions of the plasma membrane supported by nine microtubule doublets. When flagella grow, they do so by addition of new tubulin at the distal end [3]. Assembly of tubulin at the distal end depends on a motile process called intraflagellar transport or IFT [4–8]. As shown in figure 1*a*, IFT is a motile process in which a kinesin-2 motor moves a complex of proteins known as the IFT particle from the base of the flagellum out to the tip. The IFT particle is a multiprotein complex of polypeptides known as IFT proteins, which bind cargo and carry it out to the tip [9–14]. The IFT particles associate into linear arrays called IFT trains [15,16], which move back and forth on the flagellar microtubule doublets in a cycle of anterograde transport and retrograde transport [17]. The size of IFT trains, defined as the number of IFT particles per train, can vary from just a few particles to more than a dozen [18]. IFT is not only required for assembly of flagella, but also for their maintenance, such that when IFT is switched off, flagella immediately begin to shorten and resorb [5]. When IFT function is downregulated but not completely blocked, for example, when temperature-sensitive mutations in the IFT kinesin are grown at temperatures intermediate between fully permissive and fully non-permissive, flagella attain intermediate lengths [19]. Such results indicate that IFT plays a key role in flagellar length control, and suggest that regulation of IFT may be important for the regulation of length.

Live imaging of IFT in the unicellular green alga *Chlamydomonas reinhardtii* has allowed several features of the IFT system to be quantified, specifically, the size of IFT trains, the frequency with which IFT trains enter the flagellum and the speed of the trains. All three of these features vary with flagellar length [18,20]. Quantitative measurements including both live cell total internal reflection fluorescence (TIRF) imaging and stepwise photobleaching have shown that the size of IFT trains is a decreasing function of length, such that in short flagella, IFT trains contain a large number of IFT particles, while in longer flagella, the trains become much smaller. Frequency and speed both increase slightly in longer flagella. In terms of IFT function in maintaining steady-state length, the most important quantity is the product of the train size and the train injection frequency, since this determines the rate at which IFT particles enter the flagellum and move to the tip. We will term this quantity the IFT injection rate. Quantitative measurements clearly show that IFT injection rate decreases as a function of length, varying approximately as 1/*L* [20]. This dependence on length closely matches the observed kinetics of flagellar regeneration, during which flagella are seen to grow at a rate proportional to 1/*L* [21].

Fluorescence and biochemical analyses suggested that the quantity of IFT protein inside the flagellum is roughly independent of length [19,21], and this observation can now be understood as a consequence of the 1/*L* dependence of IFT injection. Each IFT particle moves to the tip of the flagellum and back, with a speed that depends only slightly on length. Therefore, the time spent by each particle inside the flagellum is proportional to the length. The total quantity of particles inside the flagellum is simply the product of the rate of particle entry (which scales as 1/*L*) and the time that each particle spends inside the flagellum (which scales as *L*), hence the product should be independent of length, exactly as observed. Thus, the earlier biochemical measurements showing constant IFT protein content [21] confirm the results of quantitative live cell imaging that the IFT injection rate varies as 1/*L*.

We currently do not know what mechanism creates the 1/*L* dependence of IFT injection on length. Biochemical studies have shown that the entry of IFT trains into the flagellum in *Chlamydomonas* is regulated, at least in part, by phosphorylation of the non-motor subunit of kinesin-2 [22,23], but the mechanisms that might regulate this phosphorylation as a function of length remain to be determined. We and others have proposed several theoretical models for the 1/*L* dependence [24], and have attempted to test some of these models [25,26].

One model we have considered is a ‘time of flight’ model in which IFT entry is gated by the time it takes for an IFT particle to return to the basal body after being injected into the flagellum. IFT velocity shows an approximately linear dependence on length with a shallow slope and a large intercept [18]. Consequently, the time spent travelling out to the tip and back again is roughly proportional to the length. Therefore, if some form of molecular timer was attached to the IFT particle, the elapsed time upon return of such particles would provide a measure of length that could be used to gate entry of new IFT particles. This type of model requires two molecular components, one to act as a timer and one to act as a gate for IFT entry that is dependent on the timer. For the timer, G proteins within the IFT particle [27] are potential candidate timers, but the timer could also be implemented by a phosphorylation event that occurs inside the flagellum. Indeed, there are many ways to imagine a molecular timer that could record time spent by the IFT particles in transit. Likewise, there are several candidate molecular components that could be acting as a gate to recruit or regulate IFT entry. It has been proposed that IFT entry might be gated by a nuclear-pore-like mechanism [28], and it has also been found that actin plays a role in IFT recruitment [29], and so it was proposed that an indirect measure of length such as time of flight could modulate such gating or recruitment mechanisms to allow injection of an appropriate quantity of IFT particles. In order to test the time-of-flight model without needing to specify the precise mechanism of timing or injection, the retrograde IFT velocity was reduced using a mutation in cytoplasmic dynein [30]. Under the time-of-flight model, such a reduced retrograde speed should mimic a longer flagellum, leading to reduced IFT injection, but this result was not observed, arguing that the time-of-flight mechanism is unlikely to explain the length dependence of IFT injection [25]. An alternative model that has been considered is based on the idea that the kinesin-2 motor that drives IFT must diffuse back to the base of the flagellum in order to drive further injection, and the rate at which the diffusing motors return to the base is a function of flagellar length. Computational simulation of such a model shows that it can in principle account for length regulation [26], but the model awaits experimental validation.

Regardless of the mechanism creating the 1/*L* dependence of injection on length, the fact that IFT injection decreases as length increases has potential implications for how flagellar length is regulated. We have previously reported a model in which flagellar length is determined by the balance between assembly and disassembly. The doublet microtubules of the flagellar axoneme undergo continuous disassembly at their distal ends [19,31,32], and we have found that this disassembly rate is independent of the length [19]. Because IFT is length-dependent, with a lower rate of IFT injection in longer flagella, it is proposed that the rate of assembly of new tubulin onto the tip will be reduced as flagellar length increases. As flagella elongate, the assembly rate will continue to reduce until the assembly rate equals the disassembly rate and, at this point, a steady state will be reached and further growth will cease. This steady-state solution is stable, because if the flagellum were to become too long, the disassembly rate will be higher than the assembly rate, causing the flagellum to shorten back to the correct length; whereas if the flagellum were to become too short, assembly will predominate and the flagellum will elongate back to the correct length.

This conceptual model can be rendered in simple mathematical form. If we combine the observation of length-independent disassembly with the observation of the 1/*L* dependence of IFT injection, and we further assume that the assembly rate is proportional to the rate of IFT injection (on the grounds that every injected particle will move to the tip and deliver tubulin for further assembly), we obtain the differential equation, which we have previously described [21], given here in figure 1*b*. In this equation, *D* is the rate of microtubule disassembly. The term (*A*/*L*) reflects the 1/*L* dependence of injection on rate. The constant *A* is termed the injection coefficient and is a lumped parameter that is the product of three factors. The first factor contributing to *A* is a proportionality constant that relates the injection rate, in terms of IFT particles per unit time, to the length. The second factor is a proportionality constant that describes the increase in flagellar length due to the arrival of a single tubulin cargo. The third factor is an association constant that describes the occupancy of an IFT particle with tubulin under the assumption of first-order binding with a length-independent dissociation constant. This equation thus assumes that the affinity of cargo for IFT particles is constant and not dependent on length, such that if the rate of IFT injection doubles, the rate of flagellar assembly must likewise double. The term (*P* − 2*L*) reflects the free tubulin pool, where *P* is the total quantity of tubulin in the whole cell, in length-equivalent units. When plotted in graphical form (figure 1*c*), we can visually see that this equation has a single steady-state solution. The graph shows two intersecting curves—a horizontal line that depicts the length-independent disassembly rate *D*, and a hyperbola that depicts the 1/*L* dependence of injection, under the assumption that the rate of assembly depends on the rate of injection. The two curves only intersect at a single value of length, which is thus a unique steady-state solution.

This model is able to account for a number of phenomenological observations including the ability of a cell to equalize the lengths of its two flagella [19,33] and the variation of flagellar length as a function of flagellar number [21]. However, the model rests on the assumption that flagellar assembly rate is proportional to the IFT injection rate.

Recently, an alternative model for flagellar length regulation has been proposed, in which the loading of tubulin and other cargo onto the IFT trains is the key length-regulated step, and length dependence of IFT injection itself is not viewed as being the relevant aspect of the process [11,14].

In this report, we examine the role of IFT injection and ask whether it could be sufficient to explain length regulation, or whether instead length-regulated cargo loading is a necessary component of the system. First, we examine mutations that increase IFT injection, and ask whether they produce the expected increase in steady-state flagellar length. As shown by the dashed line in figure 1*c*, an increase in the injection versus length curve will cause the two curves to intersect at a new, longer length. Thus, mutants that increase IFT injection should lead to increased length to a degree that is predictable by the equation in figure 1*b*. If they do not, such a result would strongly suggest that there must be an additional length-dependent step, possibly cargo loading, that would be required to explain length control. To explore this latter possibility, we develop a simple mathematical model, combined with quantitative data on IFT train size as a function of length, to ask how the published measurements of tubulin cargo loading might be predicted to vary with length under the assumption that tubulin occupancy of individual IFT particles is not length-dependent. Deviations from this prediction would provide a quantitative measure of length-regulated cargo loading. Our results indicate that length-dependent IFT injection is not entirely sufficient to explain the length increase seen in the mutants, but also that length-dependent tubulin cargo loading may play even less of a role at least in a quantitative sense.

## 2. Testing the role of length-regulated intraflagellar transport injection in length control

### (a) Increased intraflagellar transport injection in *lf* mutants

In order to ask whether regulation of IFT plays a central role in length regulation, our approach is to analyse mutants of *Chlamydomonas* that show increased IFT as a function of length, and ask whether these mutants lead to the predicted increase in steady-state length. We previously reported that mutations in the LF4 gene showed a dramatic increase in IFT injection [20]. Because the *lf4* mutant also showed long flagella, we hypothesized that other mutants with a similar long-flagella phenotype might show increased IFT.

Currently, there are five genes known in *Chlamydomonas* which, when mutated, cause flagella to become abnormally long [34–40]. However, in no case is the mechanistic function of these genes understood. The LF proteins differ in terms of their cellular localization. The first three, LF1, 2, and 3, appear to encode components of a cytoplasmic protein complex known as the ‘lengthosome’ [41], while the LF4 protein is localized both inside the flagellum and also in the cell body [37,42]. Another reported difference is the effect of the mutations in flagellar regeneration, with *lf1*, *lf2* and *lf3* mutations showing less effective regeneration after flagellar severing, while *lf4* mutants do not show a similar impairment. Double mutants between any of the three genes encoding the lengthosome components cause complete loss of flagella, and this phenotype is suppressed by the *lf4* mutation [36]. The basis of these genetic interactions remains unclear. LF2 and LF4 encode kinases, but none of the substrates are yet known, nor are upstream regulatory inputs controlling the activity of these kinases well characterized. At least in the case of LF4, kinase activity is required for its length-regulating function, and the activation loop is phosphorylated during flagellar growth, consistent with the idea that some signal is triggering LF4 kinase activity in short, growing flagella [42]. Deletion alleles of LF3 cause a short-flagella phenotype, while by contrast deletion alleles of LF4 cause long flagella. There are thus dramatic differences at the genetic and localization level between the lengthosome genes LF1, 2 and 3, compared with LF4. For this reason, it was of interest to determine whether increased IFT injection was a common feature of *lf* mutants, or just something observed in the *lf4* mutant.

**Figure 2.**
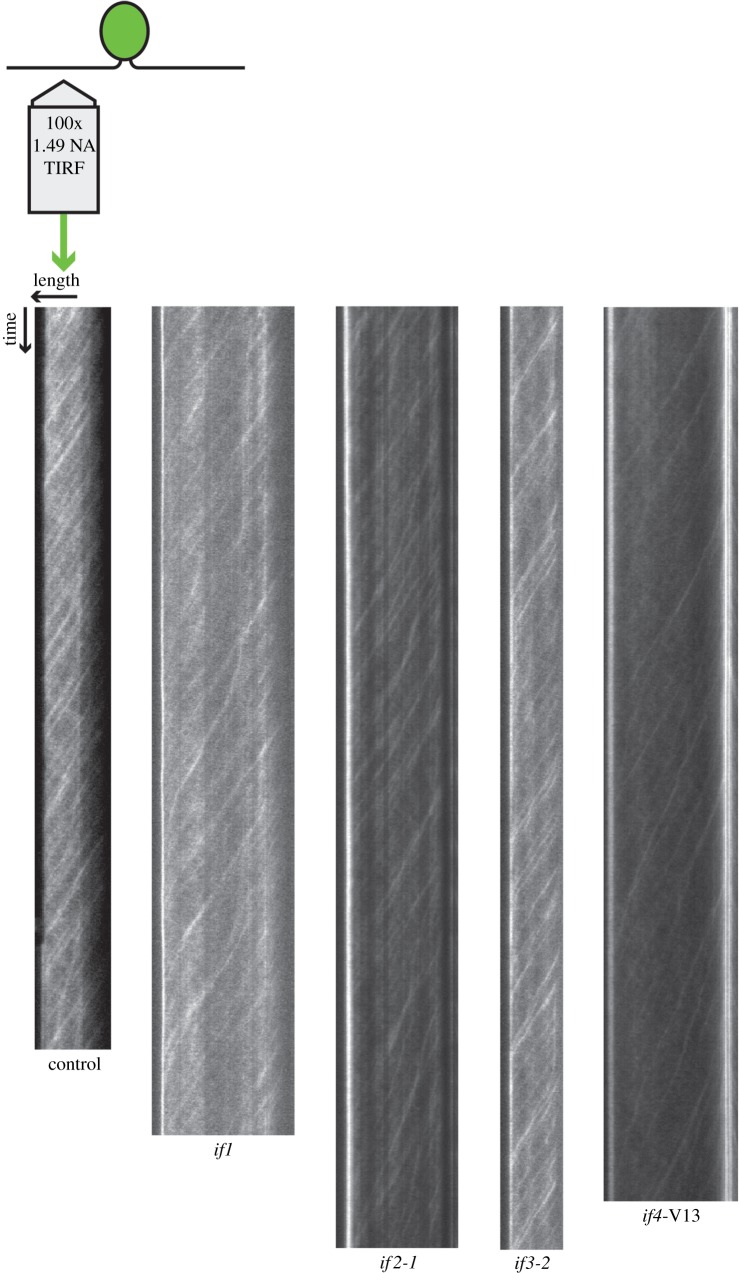
Measuring IFT injection in *lf* mutant strains. *Chlamydomonas* cells are adhered to coverslips and imaged using TIRF microscopy. Time-lapse images of flagella are stacked to produce kymographs as shown. The length of kymographs varies from cell to cell depending on how long the cell remains attached to the coverslip.

To test whether any of the existing *Chlamydomonas lf* mutants might have increased IFT in their flagella, we constructed strains expressing GFP-tagged KAP (FLA3) in each of three *lf* mutant backgrounds (*lf1, lf2* and *lf4*) as described in the electronic supplementary material, and confirmed that the long-flagella phenotype seen in parental strains was still present in the GFP-KAP-expressing derivatives. We then imaged IFT using TIRF microscopy as previously described [18,20] and generated kymographs for each cell by stacking up images of flagella as a function of time (figure 2). Within such a kymograph, the processive motion of an IFT train is revealed as a diagonal streak or trace running from the base to the tip of the flagellum.

IFT within a kymograph can be described by three parameters: speed, which is the average rate at which an IFT train moves out to the tip and is calculated by the slope of the traces in the kymographs; frequency, which is the number of times a new IFT train leaves the base of the flagellum and moves out towards the tip, per unit time; and magnitude, which is the intensity of GFP fluorescence in the trace. Magnitude, also referred to as size, is proportional to the number of IFT particles in the train. We extracted these three quantitative parameters from each kymograph using an automated algorithm previously reported [20,43]. This image analysis algorithm works by projecting the kymograph onto a single axis, testing a range of directions and choosing the direction that maximizes the difference between the highest and lowest projected intensities. The idea is that because IFT particles show highly processive motion at constant speed, they result in straight lines in the kymograph and since the speed of different IFT trains are highly similar, an axis can be chosen that aligns to the linear traces of the trains, such that when the kymograph is projected along this axis, the result will be a plot in which traces in the kymograph correspond to peaks in the plot. Speed is extracted from the angle of projection, frequency of injection is extracted from the size of the gaps between peaks, and injection magnitude (train size) is extracted from the area under each peak. The injection rate is then calculated as the product of the frequency and the magnitude, and it is this quantity that we will focus on.

The reason to focus on injection rate is that speed has no effect on flagellar growth rates, since every particle that enters will reach the tip. Frequency and size both affect growth rates, but their combined effect is entirely described by their product, the injection rate. Therefore, injection rate is the key factor in testing the role of IFT in length regulation.

**Figure 3.**
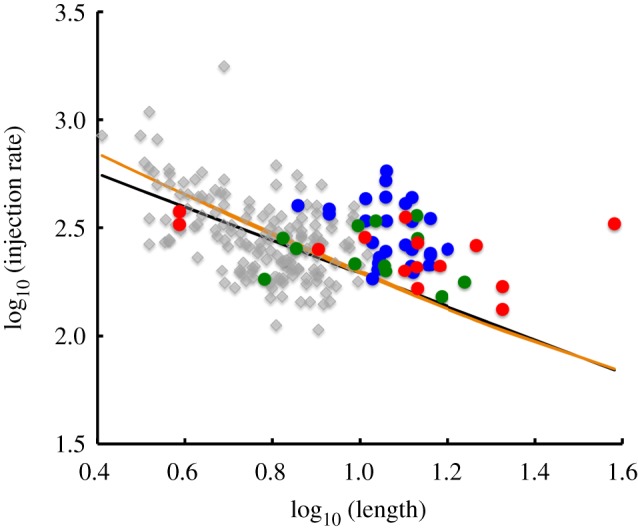
Comparing IFT injection in long-flagella mutants versus wild type. Graph shows IFT injection rate, calculated from kymograph data, plotted as a function of flagellar length. Each marker represents a different flagellum. Grey, wild type; blue, *lf1*; green, *lf2*; red, *lf4*. Black line, best-fit power-law relation for wild-type data; gold line, best-fit 1/*L* relation. (Online version in colour.)

The injection rate is plotted versus length for wild-type and *lf* mutants in figure 3. It is difficult to compare the *lf* results with wild type because they cover different ranges of length. To facilitate this comparison, we calculated the best-fit trend line for the wild-type cells, plotted as a black line in figure 3. As discussed above, we previously found, in analyses of larger numbers of wild-type cells, that the injection rate is proportional to 1/*L*, which would correspond to a straight line with a slope of −1 on this plot. Because the best-fit line in this particular dataset happened to be different from our previously measured best-fit slope of −1, we also included a best-fit line that was constrained to have a slope of −1, shown as a gold line in figure 3. For all three *lf* mutants, it is clear that the data lie above either trend line, indicating increased IFT injection rates compared to wild type. This is also evident without the trend line when considering cells that happen to be in a wild-type length range.

We conclude that *lf1*, *lf2* and *lf4* mutants all appear to alter the dependence of injection rate on length, such that at any given length, an increased rate of IFT injection is seen in the mutants.

### (b) Is steady-state length of *lf* mutants consistent with increased intraflagellar transport?

In order to ask whether the *lf* mutants change the injection rate enough to account for the increase in length, the first step is to quantify the change in injection rate. To do so, we note that on a log–log scale, the difference in log of injection rate (listed in table 1 as average difference from best-fit trend line) is the log of the fold change in injection rate. We therefore convert the difference in the log of injection rates into a fold change in injection. Because our previous studies [20] have suggested that with larger numbers of wild-type cells, the best-fit line has a slope of −1, we also computed the fold change in injection based on the deviation from the ideal 1/*L* trend line. Both sets of results were similar and both are given in table 1.

**Table 1.** Changes in length and injection rate in long-flagella mutants.

strain	length	fold change in length	average difference from best-fit line	fold change in injection (best-fit line)	average difference from trend line with slope −1	fold change in injection (slope = −1)
wt	9.9 ± 2.9	n.a.	0.03 ± 0.07	n.a.	−0.03 ± 0.16	n.a.
*lf1*	17.9 ± 2.5	1.8	0.24 ± 0.14	1.7	0.25 ± 0.14	1.8
*lf2-1*	16.7 ± 4.3	1.7	0.18 ± 0.12	1.5	0.19 ± 0.11	1.5
*lf4*	19.9 ± 8.4	2.0	0.22 ± 0.18	1.7	0.23 ± 0.17	1.7

The data in table 1 indicate that the fold changes in injection rate are comparable to the fold changes in steady-state length. However, it would be misleading to compare these two changes, because our mathematical model indicates that steady-state length is not linearly proportional to the injection rate coefficient *A*. We therefore need to derive a relation that predicts the fold change in length produced by a given fold change in injection coefficient *A*. To do so, we first find the steady-state solution to the differential equation of flagellar length dynamics by setting the rate of change to zero, as follows:

2.1 $$\frac{dL}{dt}=A\frac{\left(P-2L\right)}{L}-D=0,$$

where *L* is the length, *P* is the total size of the flagellar precursor pool (tubulin) expressed in length equivalents, *D* is the rate of flagellar disassembly and *A* is the injection coefficient. Solving for *L*, we find

2.2 $$L=\frac{P}{2+(D/A)}.$$

We can then write the expected ratio of flagellar lengths in a mutant (*L_M_*) compared to the flagellar length in wild-type cells (*L*_wt_), given that the injection coefficients for the two cases are *A_M_* and *A*_wt_, respectively

2.3 $$\frac{L_{M}}{L_{\mathrm{wt}}}=\frac{\left(2+(D/A_{\mathrm{wt}})\right)}{\left(2+(D/A_{M})\right)}.$$

Because the numerator and denominator have a constant term 2 added to the ratio *D*/*A*, it is evident that fold change in steady-state flagellar length (*L_M_*/*L*_wt_) will always be less than the fold change in IFT injection rates as measured by the ratio of the injection coefficient *A* between mutant and wild type. If *D*/*A* is much larger than 2, then the fold change in lengths will become close to the fold change in injection coefficients, but never exceed it. We previously estimated the ratio *D*/*A* for wild-type cells by fitting the dependence of flagellar length as a function of flagellar number in mutants with variable numbers of flagella [21]. That analysis suggested that the ratio *D*/*A* was in the range 6–8. Taking the high end of this range, and dividing *D*/*A* by the fold increase in injection coefficient from table 1, we obtain the predicted fold increases in length listed in table 2.

**Table 2.** Prediction for fold change in length based on change in injection rate.

strain	length	fold change in length	predicted fold change in length (best-fit line)	predicted fold change in length (line with slope −1)
*lf1*	17.9 ± 2.5	1.8	1.5	1.5
*lf2-1*	16.7 ± 4.3	1.7	1.4	1.4
*lf4*	19.9 ± 8.4	2.0	1.5	1.5

The conclusion of table 2 is that while all the *lf* mutants are predicted to have increased length, the predicted length increase is not quite as large as that actually seen (for example, 1.5 versus 1.8 for *lf1*). It is possible that some of this discrepancy is due to measurement error, but it may also reflect the fact that our mathematical model is highly simplified and likely ignores many important aspects of the system. One such aspect will be considered in the next section.

## 3. Testing the role of length-regulated cargo-binding in length control

Given that the increased rate of IFT injection does not fully account for the increased length seen in the *lf* mutants, it is likely that the highly simplified model we have presented may still be missing one or more length-dependent terms that we do not account for. The recent demonstration of apparent length-dependent tubulin loading on IFT trains could be one such missing factor [14]. In order to incorporate regulated cargo loading into our model, we need a way to quantify the extent to which cargo loading is regulated as a function of length. To do so, we first must determine the null hypothesis, that is, what is our prediction concerning the occupancy of tubulin on IFT trains in the case that tubulin binding is not affected by length at all. We can then compare this prediction to the published data of Craft *et al*. [14], and the deviation from the prediction will tell us the extent to which cargo loading is length regulated.

**Figure 4.**
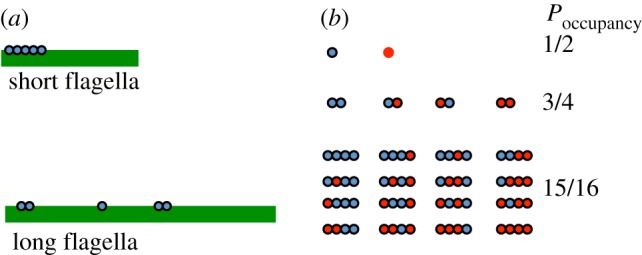
Prediction of apparent length dependence for cargo loading. (*a*) Extensive prior analysis of IFT dynamics has shown that the number of IFT particles per IFT train is a decreasing function of flagellar length. (*b*) Example of *P*_occupancy_ per train as a function of train size, assuming a constant probability of cargo-binding per IFT particle. As the number of particles per train increases, there are more sites for the cargo to bind, hence *P*_occupancy_ decreases even though there is no change in the occupancy of individual IFT particles.

We therefore seek a theoretical model that can predict the outcome of the tubulin occupancy measurements in the absence of length-regulated binding. In their reported results, Craft *et al.* imaged tubulin being transported by IFT in *Chlamydomonas* flagella, and reported ‘occupancy’ as the fraction of IFT trains that had an associated tubulin signal. In order to think about such results, we recall the distinction between IFT trains and IFT particles discussed in the Introduction. ‘IFT particle’ refers to the individual protein complexes made up of IFT proteins, while ‘IFT train’ refers to the linear array of particles that move as a group within the flagellum. Because tubulin occupancy has been determined as a function of IFT trains, while tubulin binding is a function of individual IFT particles, it is critical to take into account the fact that the number of particles per train is a function of length. Figure 4*a* diagrams the results of our prior experiments [18] in which we showed, both by quantitative TIRF microscopy and by stepwise photobleaching, that IFT trains consist of fewer and fewer IFT particles as the flagellar length increases. Thus, short flagella have long trains with many IFT particles, while long flagella have short trains with few IFT particles.

We can see how train size can affect measurements of tubulin occupancy by considering the hypothetical example diagrammed in figure 4*b*. In this simple example, we assume that the binding affinity of tubulin for IFT particles is such that each particle has a 50% chance of having a tubulin bound to it. For very short trains consisting of a single particle per train, the probability of observing tubulin signal associated with the train is thus 50%. For slightly larger trains consisting of two particles, the fact that the train now contains two binding sites for tubulin means that there are four possible binding states, three of which would be detected as a tubulin signal associated with the train. The measured probability of occupancy would thus increase to 75%, even though the binding affinity of tubulin to an individual particle has not changed. This is simply a consequence of having more binding sites per train. Going a step further to a larger train with four particles, the probability of occupancy increases to 15/16, and one can see that as the train size continues to increase, the probability of observing a tubulin signal associated with the train will increase to close to 100%, even though the probability that any given particle has a bound tubulin is still only 50%.

We can formalize this model as follows. Let *N* be the number of IFT particles per IFT train, where each of the *N* particles contains a binding site for tubulin. If *P*_bound_ is the probability that one of these particles has a tubulin dimer bound to it, then the probability that the train appears to be ‘empty’, that is, lacking a bound tubulin, is

3.1 $$P_{\mathrm{empty}}=(1-P_{\mathrm{bound}}{)}^{N},$$

which is simply the product of the individual probabilities that each of the *N* particles does not have a tubulin dimer bound to it. On a log scale, we obtain

3.2 $$\log P_{\mathrm{empty}}=N\log(1-P_{\mathrm{bound}}).$$

**Figure 5.**
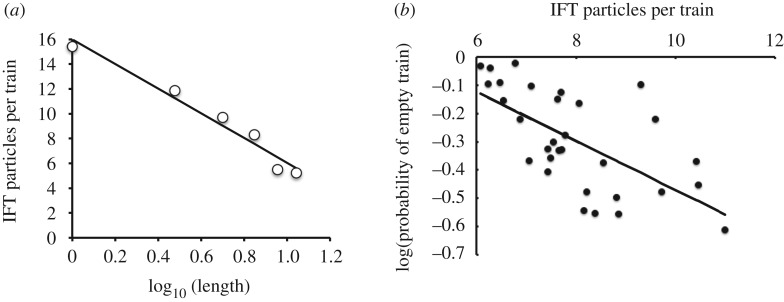
Comparison of measured tubulin cargo loading with theoretical model. (*a*) Relation between flagellar length and train size based on data from Engel *et al.* [18]. Line shows the best-fit line *N* = 16–9.96 log_10_*L*. (*b*) Predicted train occupancy in the absence of length-dependent cargo loading. Line is obtained from the theoretical analysis presented in the text. Data points were taken directly from published measurement of tubulin occupancy of IFT trains as a function of flagellar length in *Chlamydomona*s ([14] figure 3*g*), and re-plotted here as a function of train size, inferred for each data point based on flagellar length using the fit line in (*a*).

Thus, if we plot the log of the probability of a train being empty versus the number of particles, we should observe a straight line. The published data from Craft *et al*. [14] report train occupancy as a function of length, not of *N*, so in order to match their results with equation (3.2), we need a way to predict *N* for a given *L*. Figure 5*a* provides a plot of average train size *N* versus flagellar length *L*, based on our previously published results [18]. It can be seen from this plot that *N* is well fit by a linear function proportional to the log of the flagellar length. Other functions might fit the relation between *N* and *L* just as well, but for our purposes, here, we simply use the logarithmic relation as an Ansatz that allows us to make a prediction about observed tubulin occupancy. Using the best-fit line from figure 5*a*, we converted the lengths for each data point in Craft *et al.* into an equivalent train size *N*, and then re-plotted the occupancy data in figure 5*b*.

As shown in figure 5*b*, the actual measurements of *P*_empty_, as reported by Craft *et al*. [14], are well fit by a straight line as predicted from equation (3.2). This result suggests that in fact tubulin binding to IFT particles may not be length-dependent, and that the reported length dependence of tubulin loading onto trains may simply be a result of the decreasing number of particles per train in longer flagella. In order to definitively say whether tubulin loading is, or is not, length-dependent, it will be necessary to quantify the amount of tubulin per IFT particle. This has been done with non-tubulin cargo [11] but is more challenging for tubulin because the signal is less intense. It is to be hoped that in the future, brighter tubulin constructs will allow this point to be addressed.

## 4. Discussion and outlook

Our main experimental result is that of the three *lf* mutants analysed, all three showed increased IFT injection, possibly suggesting that the regulation of IFT injection may be a key role of the LF gene products. It is notable that in all three cases, injection still appears to be length-dependent, indicating that IFT is still being regulated as a function of length. Concerning previous studies, our results on increased IFT injection in *lf4* mutants are consistent with our previously published results [20] and with the reports of Wang *et al.* [42] that IFT protein quantity is increased in flagella of *lf4* null mutants. Live cell differential interference contrast (DIC) imaging was previously used to analyse quantitative parameters of IFT in a panel of mutants [17], but no *lf* mutants were analysed in that study, and in any case, DIC does not provide information on train size. Prior biochemical studies [44] indicated increased IFT protein within the flagella of *lf3* null mutants, but those studies are difficult to interpret because the *lf3* allele used caused flagella to be abnormally short, not long, so loading equal total protein per well, as done by Tam *et al*. is expected to show increased IFT protein for short flagella simply by virtue of the fact that IFT protein content tends to be length-independent. Our imaging-based results provide a more direct demonstration that *lf* mutants beyond *lf4* also affect IFT injection.

Our theoretical analysis of these results suggests that the increased injection of IFT in the *lf* mutants is able to explain part of the increase in length, indicating that length-dependent regulation of IFT is clearly playing a functional role in the length control system. On the other hand, the predicted change in length based on the increased injection rate is smaller than the length change that is actually observed in the mutants, possibly suggesting that our model as currently formulated may be missing one or more important aspects of the system. We had suspected that the missing piece might be length dependence of tubulin loading; however, the results of figure 5 suggest that existing data are insufficient to determine whether tubulin loading is length-dependent. It is possible that if tubulin quantity per particle could be measured, it would show a length dependence.

Our analysis has focused on the effect that the *lf* mutations have on IFT, but it is certainly possible that other aspects of the length control system could also be altered in the long-flagella mutants, and that these other alterations contribute to the large increase in length that is seen. For example, if an *lf* mutation were to decrease the disassembly rate, this would lead to increased length above and beyond that predicted based solely on the increase in IFT. One could also imagine that the *lf* mutants might affect cargo loading or precursor protein synthesis. In our present analysis, we focused on the increase in IFT injection, but future studies will need to be more comprehensive.

Our analysis has focused entirely on mutations that cause abnormally long flagella to form. A number of mutations have been characterized that result in abnormally short flagella, and if some of these mutations resulted in decreased IFT, they could provide a valuable alternative source of data for testing the role of IFT in length control. Currently, however, our TIRF-based approach does not allow us to reliably measure IFT in very short flagella, for two reasons. First, short flagella do not stick very well to the glass coverslip. Second, because the cell body is spherical, the most proximal portion of the flagellum is unable to lie flat in the TIRF field, making it difficult to quantify IFT in that region. The solution will be to move away from TIRF imaging and mount cells in such a way that the entire flagellum can be imaged in a single plane. Technology for mounting cells in microfluidic devices has been reported [33].

The work discussed here focuses on *Chlamydomonas* flagella as a model system. This system has many advantages for the study of IFT and its regulation, such as a large set of pre-existing, well-characterized genetic mutations, the rapid and convenient culture of *Chlamydomonas* cells, and the ease of imaging *Chlamydomonas* flagella by TIRF microscopy. However, the IFT system and indeed the entire machinery of the axoneme is highly conserved between algae and mammals, making it likely that results obtained in *Chlamydomonas* will also apply in human cells. This is of potential importance, given that the length of primary cilia is sometimes altered in human ciliopathies.

## Supplementary Material

Supplementary methods and tables

## Supplementary Material

Data files

## Data accessibility

This article has no additional data.
