# Supplementary material for: Testing the role of intraflagellar transport in flagellar length control using length-altering mutants of Chlamydomonas
Source: Philos Trans R Soc Lond B Biol Sci. 2019 Dec 30;375(1792):20190159. doi: 10.1098/rstb.2019.0159 (PMC7017341; doi:10.1098/rstb.2019.0159)
Supplement: Supplementary methods and tables [file rstb20190159supp1.docx]

**Supplementary Material**

The following provides details concerning experimental methods.

***1. Strains construction***

Starting strains are described in Table S1 and were either obtained from the *Chlamydomonas* Stock Center or else were generously provided by the individuals indicated in the table. Using these strains, we constructed haploid *lf* mutant strains expressing GFP-tagged KAP subunit of kinesin-2, as indicated in Table S2. In order to avoid significant quantity of unlabeled KAP subunit, all strains were mutant for FLA3, the gene encoding KAP, using an allele that produces low quantities of the protein. Strains were constructed using standard mating and tetrad dissection procedures except that in cases that mated poorly, mating was induced using IBMX and dibutyryl cyclic AMP. All methods are described in the Chlamydomonas Sourcebook (S1). Genotypes were verified using PCR detection of all relevant alleles using primers described in Table S3.

***2. media and cell growth***

Strains were maintained on TAP agar plates. Prior to imaging, cells were grown overnight in 5 mL of M1 media in 15 mL sterile test tubes in a roller drum at 21C in constant light.

***3. TIRF analysis of IFT***

Imaging of IFT in live cells by TIRF microscopy was performed exactly as described in (20). Briefly: cells were allows to adhere to a glass coverslip within a droplet of media contained in a ring of Vaseline. Cells were then imaged with a Nikon te2000 microscope with a 100x 1.49 n.a. lens in TIRF mode using a 491 nm laser, with images collected at a frame rate of 29.7 fps. Cells were imaged as they were found in the field of view, and were not selected based on the length of their flagella, in order to obtain an unbiased measure of IFT. As noted in Figure 2, the length of time that a flagellum could be measured depended on how long the cell remained attached to the coverslip. We chose not to select image series with an artificially imposed time range, nor did we truncate time-lapse series to make them all the same length, since we cannot rule out meaningful variation as a function of time during collection. In any case we did not observe any systematic difference in the time attached to the coverslip between the various lf mutants. We also avoided chemical treatments that paralyze flagella in case these might affect IFT. IFT injection rate was computed from the kymograph data as previously described (20,43). A conceptual discussion of the algorithm is provided in Section 2.1 of the main text, but details of implementation can be found in (20).

**Supplementary References**

S1. Harris EH. 1989. *The Chlamydomonas sourcebook : a comprehensive guide to biology and laboratory use*. Academic Press, San Diego. xiv, 780 pp.

S2. Mueller J, Perrone CA, Bower R, Cole DG, Porter ME. 2005. The FLA3 KAP subunit is required for localization of kinesin-2 to the site of flagellar assembly and processive anterograde intraflagellar transport. *Mol. Biol. Cell* **16**, 1341-1354.

**Table S1. Starting strains used for construction.** cc numbers denote strains obtained from the Chlamydomonas genetics center.

| **strain** | **source** | **references** |
| --- | --- | --- |
| fla3^ts^::KAP-GFP | Dr. Mary Porter | S2 |
| lf1 | cc1678 | 34; 35; 36; 38 |
| lf2-1 | cc803 | 3435; 36; 39 |
| lf4-V13 | Dr. Gregory Pazour | 36; 37 |

**Table S2.** *Chlamydomonas* strains created for this work.

| **strain created** | **parental strain mating type -** | **parental strain mating type +** |
| --- | --- | --- |
| KAPGFP Bx1 mt- | KAPGFP | cc125 |
| lf1; fla3^ts^; KAPGFP | cc1678 | KAPGFP Bx1 |
| lf2-1; fla3^ts^; KAPGFP | KAPGFP | cc803 |
| lf4-V13; fla3^ts^; KAPGFP | KAPGFP | lf4-V13 |

**Table S3.** Primers used in strain verification.

| allele | primers | band size (bp) | method  of ID | digest results |
| --- | --- | --- | --- | --- |
| fla3^ts^ | fla3-1 CAPS-F2  CTGCTGCAGGACAAGAACAA | 520 | XhoI | WT:  520  fla3-1:  268+252 |
|  | fla3-1 CAPS-R2  TAGTCCACCTCCGTCTCGTC |  |  |  |
| lf1 | LF1-F1  TCTGCATCGTGCAGTAAGCACT | 321 | sequence | NA |
|  | LF1-R1  TATGTCAATACGGAGCGTGGCCT |  |  |  |
| lf2-1 | LF2-1-F1  GGCGCAAATACTTGTCAGAGCACA | 536 | sequence | NA |
|  | LF2-1-R1  TATCGGCACACCTAACTTCCGTCA |  |  |  |
| lf4 | LF4-F5  CTGCTGCTGTCATGTCGTTT | 161 | presence or  absence of  the band | NA |
|  | LF4-R5  ATCTTACTCGGCCAGCTCAA |  |  |  |
| MT plus | MT P2 forward  GCTGGCATTCCTGTATCCTTGACGC | 423 | presence or  absence of  the band | NA |
|  | MT P2 reverse  GCGGCGTAACATAAAGGAGGGTCG |  |  |  |
| MT minus | MT M3 forward  CGACGACTTGGCATCGACAGGTGG | 689 | presence or  absence of  the band | NA |
|  | MT M3 reverse  CTCGGCCAGAACCTTTCATAGGGTGG |  |  |  |

NA indicates 'not applicable'.
